# Centriolar Protein C2cd3 Is Required for Craniofacial Development

**DOI:** 10.3389/fcell.2021.647391

**Published:** 2021-06-15

**Authors:** Ching-Fang Chang, Kari M. Brown, Yanfen Yang, Samantha A. Brugmann

**Affiliations:** ^1^Division of Developmental Biology, Department of Pediatrics, Cincinnati Children’s Hospital Medical Center, Cincinnati, OH, United States; ^2^Department of Pediatrics, University of Cincinnati College of Medicine, Cincinnati, OH, United States; ^3^Division of Plastic Surgery, Department of Surgery, Cincinnati Children’s Hospital Medical Center, Cincinnati, OH, United States; ^4^Shriners Children’s Hospital, Cincinnati, OH, United States

**Keywords:** primary cilia, ciliopathies, C2cd3, craniofacial development, neural crest

## Abstract

The primary cilium is a ubiquitous, microtubule-based cellular organelle. Primary cilia dysfunction results in a group of disorders termed ciliopathies. C2 domain containing 3 centriole elongation regulator (C2cd3), encodes a centriolar protein essential for ciliogenesis. Mutations in human C2CD3 are associated with the human ciliopathy Oral-Facial-Digital syndrome type 14 (OFD14). In order to better understand the etiology of ciliopathies including OFD14, we generated numerous murine models targeting C2cd3. Initial analysis revealed several tissue-specific isoforms of C2cd3, and while the loss of C2cd3 has previously been reported to result in exencephaly, tight mesencephalic flexure, pericardial edema, abnormal heart looping and a twisted body axis, further analysis revealed that genetic background may also contribute to phenotypic variation. Additional analyses of a conditional allelic series targeting C-terminal PKC-C2 domains or the N-terminal C2CD3N-C2 domain of C2cd3 revealed a variable degree of phenotypic severity, suggesting that while the N-terminal C2CD3N-C2 domain was critical for early embryonic development as a whole, there was also a craniofacial specific role for the C2CD3N-C2 domains. Together, through generation of novel models and evaluation of C2cd3 expression, these data provide valuable insight into mechanisms of pathology for craniofacial ciliopathies that can be further explored in the future.

## Introduction

The primary cilium is a cellular organelle comprised of a microtubule-based axoneme extending from the cell surface, and a basal body that resides internally at the base of axoneme. Defects in the structure or function of the ciliary complex result in a class of diseases called ciliopathies. Ciliopathies affect as many as 1 in 800 people. Currently, there are 26 known ciliopathies, 25 predicted ciliopathies and another 400 human diseases considered possible ciliopathies that have yet to be classified (Schock and Brugmann, 2017). Although ciliopathies often present with pleiotropic phenotypes including renal disease, retinal degeneration, obesity, skeletal dysplasia, and craniofacial anomalies (Reiter and Leroux, 2017), 30% of ciliopathies are primarily classified by their craniofacial phenotypes. Craniofacial ciliopathies are most frequently defined by the combinatorial presentation of cleft lip/palate, craniosynostosis, hypertelorism, and micrognathia (Schock and Brugmann, 2017). Interestingly, among the 187 genes that are associated with known ciliopathies, 50 genes encode proteins that localize to basal bodies or centrosomes, and an additional five genes encode proteins that localize to centriolar satellites (Reiter and Leroux, 2017). Thus, understanding formation and function of the basal body is essential for gaining insights for therapeutic treatment of ciliopathies.

The basal body is a modified mother centriole within the centrosome. During G1-S phase of the cell cycle, the daughter centriole gradually loses its daughter centriole-specific proteins and acquires distal and subdistal appendages in late G2 phase. The acquisition of distal appendages and subdistal appendages marks the maturation of the mother centriole and distinguishes the mother centriole from the daughter centriole. During intracellular cilium assembly, Golgi-derived ciliary vesicles dock and fuse at the distal end of the mother centriole, a step called centriole-to-basal body transition. The ciliary axoneme is then assembled by extension of centriolar microtubules underneath the ciliary vesicular cap, while the basal body migrates to the plasma membrane. This basal body/nascent cilium complex then docks and fuses to the plasma membrane through vesicular fusion, a process mediated by distal appendages, which is followed by the further growth and maintenance of the axoneme at the cell surface (Wang and Dynlacht, 2018). The mother centriole within the basal body, the daughter centriole, pericentriolar material (PCM), and centriolar satellites comprise the centrosome, the major microtubule organizing center (MTOC) of the cell. Components localized to the cilium and/or centrosome play essential roles in the dynamics between both organelles; therefore, regulation of the two organelles are tightly linked (Joukov and De Nicolo, 2019).

Recent studies have uncovered several key regulators of centriole maturation and subsequent ciliogenesis. For example, several distal centriolar proteins including C2 domain containing 3 centriole elongation regulator (C2cd3), Oral-Facial-Digital Syndrome 1 Protein (Ofd1) and others are required for the recruitment of distal appendage proteins to the distal end of mother centriole (Singla et al., 2010; Ye et al., 2014; Kazatskaya et al., 2017). C2cd3 and Ofd1 colocalize and physically interact at the distal end of centriole to control centriolar length. Ofd1 acts as a negative regulator of centriole elongation, constraining centriole elongation (Singla et al., 2010). Conversely, C2cd3 is a positive regulator of centriole elongation, as loss of C2cd3 results in shorter centrioles and overexpression of C2cd3 produces hyper-elongated centrioles. More intriguingly, this hyper-elongated centriole can be suppressed by Ofd1 (Thauvin-Robinet et al., 2014). While several other centriolar proteins are required for centriole maturation, elongation, and uncapping (Hsiao et al., 2009; Schmidt et al., 2012; Burke et al., 2014; Kobayashi et al., 2014; Lu et al., 2015; Bhattacharyya et al., 2016), C2cd3 and Ofd1 have been a focus of study because of their association with the human ciliopathy Oral-facial-digital syndrome (OFD).

Oral-facial-digital syndromes represent a group of human ciliopathies caused by mutations to various ciliary proteins. OFDs can be characterized by facial, oral and digital malformations. To date, there are approximately 18 subtypes of OFD syndromes,^1^ and each subtype is classified according to the associated gene. Although affected individuals in each subtype present with largely overlapping phenotypes, they do have distinct and unique clinical features. For example, whereas patients with OFD IV display severe tibial dysplasia, patients with OFD VI display cerebellar abnormalities, and patients with OFD IX are characterized by retinal coloboma, in addition to oral, facial and digital defects. Among the subtypes in which causative genes have been identified, almost all genes express proteins which localize to the basal body (Cortes et al., 2016; Franco and Thauvin-Robinet, 2016; Bruel et al., 2017). For example, the most frequent OFD subtype, OFD I, is associated with mutations in *OFD1*, which encodes a protein localized to the basal body. Mutations in *NEK1*, a centrosomal protein responsible for centriole elongation, are associated with OFD II.

Mutations in C2CD3 are associated with OFD14. C2CD3 contains a tandem array of five classical Protein Kinase C C2 domains (PKC-C2) and a novel, divergent C2CD3 N-terminal C2 (C2CD3N-C2) domain (Zhang and Aravind, 2012). C2 domains are present in many proteins and were first discovered as calcium-dependent membrane-targeting domains in the conventional (or Ca^2+^-activated) PKC isoforms (α, β, γ) (Steinberg, 2008; Farah and Sossin, 2012); however, a variety of C2 domains that have Ca^2+^-independent membrane-targeting abilities have also been identified (Lee et al., 1999; Djordjevic and Driscoll, 2002). While the PKC-C2 domain predicts membrane-anchoring function of C2CD3, the function of the divergent C2CD3N-C2 domain remains unknown (Zhang and Aravind, 2012). Over 70% of identified OFD14 causing C2CD3 mutations are located within PKC-C2 or C2CD3N-C2 domains, further suggesting the importance for understanding the role of each domain (Boczek et al., 2018).

More recently murine and avian models for OFD14 have been used to examine C2CD3 function. *Hearty* (*Hty*), a recessive lethal mouse mutant identified by forward genetic screening, showed severe developmental defects including those of the neural tube, heart and limb (Hoover et al., 2008). The *Hty* mutation resulted in a largely truncated protein, which contained the divergent C2CD3N-C2 domain but none of the more C-terminal PKC-C2 domains. *talpid*^2^ (*ta*^2^), a naturally occurring avian mutant, was recently identified as being caused by a 19 bp deletion in exon 32 of *C2CD3* (Chang et al., 2014). Long gestational survival of *ta*^2^ has allowed for additional analyses and determination that it is a bona fide model for OFD14. Mutations in C2cd3 impair the formation and function of primary cilia, and perhaps most notably the transduction of cilia-dependent Hedgehog signaling (Hoover et al., 2008; Chang et al., 2014). Moreover, migration and differentiation of cranial neural crest cells were affected in *ta*^2^, suggesting a possible cellular etiology of OFD14 (Chang et al., 2014; Schock et al., 2015). Although *ta*^2^ is a model for human OFD14, the lack of avian genetic techniques necessitates murine models for further mechanistic and tissue-dependent study.

Here we report and characterize several novel C2cd3 mouse models, including a publicly available conditional knockout line and two novel CRISPR-targeted lines, targeting regions in the divergent C2CD3N-C2 domain or PKC-C2 domains. We identified the predominant C2cd3 isoforms in a tissue-specific manner and propose that phenotypic variability is a consequence of both tissue-specific isoforms and genetic background. In sum, data presented herein can be used not only to study the etiology of ciliopathic pathologies, but also to address the specific roles of various C2-domains during development.

## Results

### *C2cd3* Is Dynamically Expressed During Embryonic and Craniofacial Development

**FIGURE 1:**
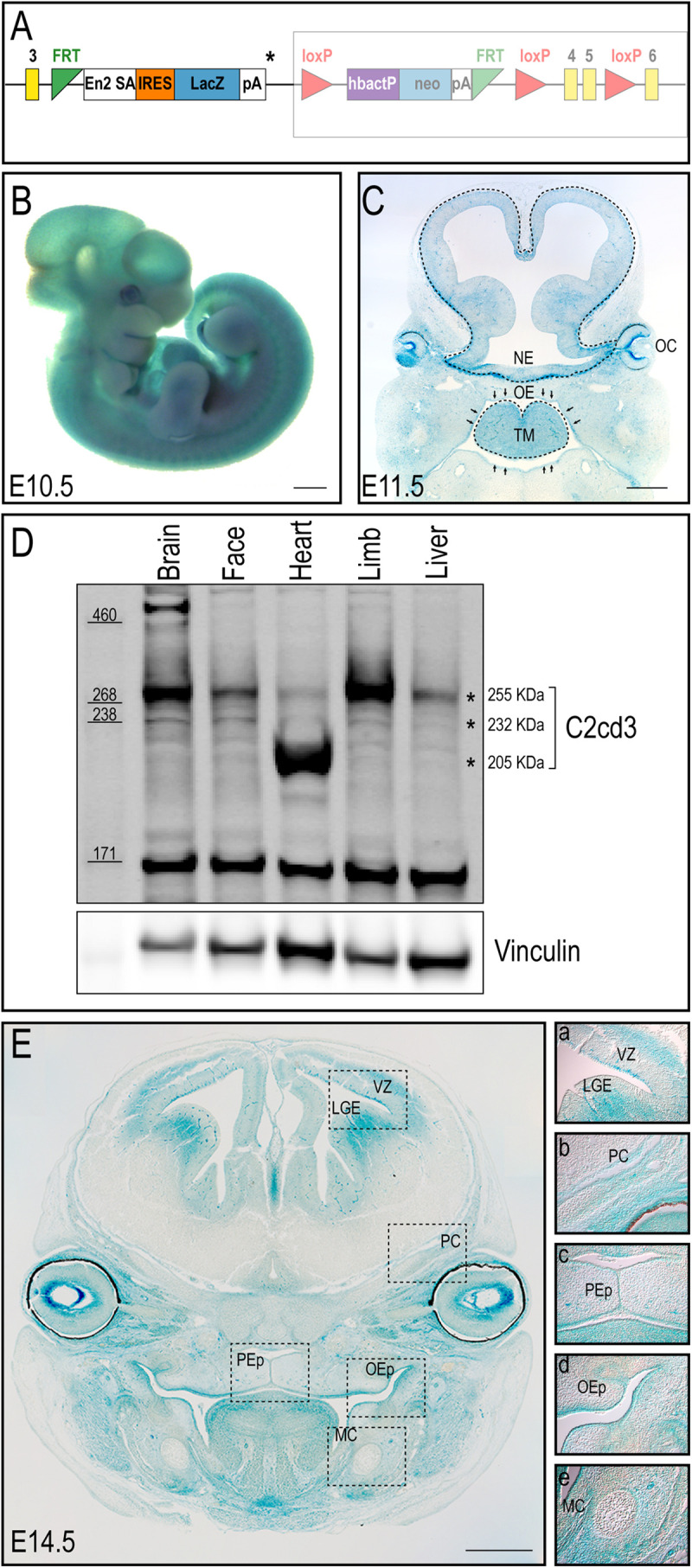
C2cd3 expression in developing mouse embryos. **(A)** Schematic of *C2cd3*^LacZ^ targeted allele. LacZ expression cassette containing a stop codon (*) was inserted after exon (yellow box) 3. The remaining, unexpressed part of the construct designed for conditional knockout is indicated in the shaded box. **(B)** Whole mount X-gal staining indicates ubiquitous C2cd3 expression in E10.5 embryos. **(C)** Frontal section of an E11.5 embryo showing robust C2cd3 expression in neural epithelium (NE, dotted line), otic cup (OC), oral epithelium (OE, arrows) and tongue mesenchyme (TM, dotted line). **(D)** Western blot of C2cd3 expression in different tissues of E11.5 wild type embryos, showing three C2cd3 isoforms (asterisks). **(E)** Frontal section of E14.5 embryo, revealing robust C2cd3 expression in the lateral ganglionic eminence (LGE) and ventricle zone (VZ) of developing neocortex, perichondrium (PC) of developing cranial bones, palatal epithelium (PEp), oral epithelium (OEp) and mesenchyme surrounding Meckel’s cartilage (MC). All scale bars: 0.5 mm.

Despite an accepted role in ciliogenesis, *C2cd3* expression has not been well documented during embryogenesis or craniofacial development. To comprehensively characterize *C2cd3* expression, *C2cd3^TM 1a(EUCOMM)Wtsi^* mouse ES cells were injected into mouse blastocysts. Recovered transgenic mice (hereafter referred to as *C2cd3*^LacZ^) expressed a *LacZ*-expression cassette after exon 3 of *C2cd3*, as well as LoxP sites flanking exon 4/5 for the option of tissue-specific gene deletion using Cre-LoxP system (Figure 1A). Heterozygous *C2cd3*^LacZ/+^ mice were viable and morphologically normal, while homozygous *C2cd3*^LacZ/LacZ^ mice died at approximately E10.5 (data not shown). Whole mount X-gal staining revealed that *C2cd3* was expressed ubiquitously at E10.5 (Figure 1B). At E11.5, however, *C2cd3* expression was more spatially distinct with the most robust expression in the neural epithelium, optic cup, oral epithelium, and tongue mesenchyme (Figure 1C). Ubiquitous, but variable levels of C2cd3 protein isoforms (255, 232, and 205 kDa) were detected in various embryonic tissues at E11.5 via Western blot analysis (Figure 1D asterisks and Supplementary Figure 1). The 255 kDa isoform was expressed at varible levels across all isolated tissues (brain, face, heart, limb, and liver) (Figure 1D, top asterisk). The 232 kDa isoform was also detected across all tissues, albiet at much lower levels (Figure 1D, middle asterisk). The 205 kDa isoform was distinct from other isoforms, as it was robustly expressed in heart tissue (Figure 1D, bottom asterisk). While protein prediction tools predicted an additional 214 kDa isoform, we were unable to detect it due to the lack of the C-terminal epitope which the antibody recognizes (Supplementary Figure 1). *C2cd3* expression was maintained later in development in both epithelial and mesenchymal tissues, including the lateral ganglionic eminence and ventricular zone of developing neocortex, perichondrium of developing cranial bones, palatal epithelium, oral epithelium, and mesenchyme surrounding Meckel’s cartilage at E14.5 (Figure 1E). Together, these results suggested that *C2cd3* expression was dynamic throughout embryonic and craniofacial development. We next sought to examine the consequences of impaired *C2cd3* expression.

### *C2cd3* Mutants Present With Multiple Embryonic Defects

**FIGURE 2:**
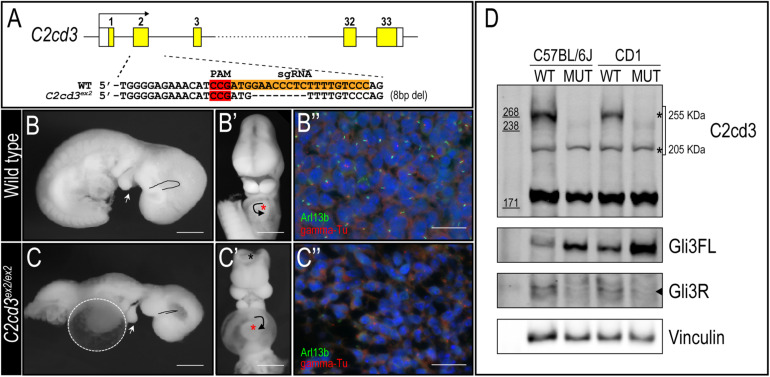
*C2cd3^ex2/ex2^* mutants present with phenotypes indicative of a ciliopathy. **(A)** Schematic of CRISPR-targeted 8 bp deletion in *C2cd3* at exon 2 which results in a premature stop codon. **(B,C)** Lateral and **(B′,C′)** frontal views of E9.5 wild-type and *C2cd3^ex2/ex2^* embryos. *C2cd3^ex2/ex2^* embryos exhibit pericardial edema (white dashed line), tight mesencephalic flexure (black line), mandibular hypoplasia (white arrowhead), abnormal heart looping (black arrow), and exencephaly (black asterisk). The heart is labeled with red asterisks. **(B′′,C′′)** Axonemal (Arl13b) and basal body (gamma-tubulin) immunostaining of E9.5 embryos in wild-type and *C2cd3^ex2/ex2^* embryos. Scale bars for panels **(B–C′)**: 0.5 mm; **(B′′–C′′)**: 50 μm. **(D)** Western blot of E9.5 wild-type and *C2cd3^ex2/ex2^* whole embryos showing deletion of the 255 kDa isoform in both C57BL/6J and CD1 backgrounds. The ratio of Gli3FL/Gli3R is increased in *C2cd3^ex2/ex2^* compared to wild-type.

To better understand the biological function of *C2cd3*, we utilized CRISPR-mediated genome editing to generate an 8 bp deletion in exon 2 that resulted in a premature stop codon (henceforth referred to as *C2cd3^ex2^*) (Figure 2A). *C2cd3^ex2/ex2^* mutants were embryonic lethal at approximately E10.5 and presented with phenotypes similar to those previously reported in *Hty* mutants (Hoover et al., 2008). Mutants were present in Mendelian ratios (Table 1A) and 90% of mutants exhibited exencephaly and a twisted body axis (Figures 2B–C and Table 1B). In addition, 30% of *C2cd3^ex2/ex2^* mutants presented with pericardial edema, abnormal heart looping, and a tight mesencephalic flexure (Figures 2B–C′ and Table 1B). Compared to the previously reported *C2cd3 Hty* mutants, which commonly presented with twisted body axis, pericardial edema, and tight mesencephalic flexure, the most common phenotypes in *C2cd3^ex2/ex2^* mutants were exencephaly and twisted body axis. Although polydactyly was also observed in *Hty* mutants at E12.5, we were unable to assess this phenotype in *C2cd3^ex2/ex2^* mutants because they did not survive past E10.5. To confirm that *C2cd3^ex2/ex2^* mutants failed to extend primary cilia, immunostaining for Arl13b (ciliary axoneme) and gamma-tubulin (basal body) was performed. Relative to the facial mesenchyme of wild-type embryos, *C2cd3^ex2/ex2^* mutants lacked Arl13b while maintaining gamma-tubulin staining, indicating that the extension of primary cilia was abolished but the basal body remained intact (Figure 2B′′,C′′). Thus, *C2cd3^ex2/ex2^* mutants lacked cilia and presented with phenotypes indicative of a ciliopathic model.

TABLE 1. Phenotypic presentations of C2cd3 mutants. **(A)** Total number of wild-type, *C2cd3^ex2/+^*, and *C2cd3^ex2/ex2^* embryos, *n* = number of litters on CD1 and C57BL/6J backgrounds. **(B)** Percentages of phenotypes present in *C2cd3^ex2/ex2^* mutant embryos.

**TABLE 1A:** Number of wild-type, *C2cd3 ^ex2/+^*, and *C2cd3 ^ex2/ex2^* embryos on CD1 and C57BL/6J backgrounds.

Background	Total	*n*	Wild-type	Het	Mutant
Outcrossed CD1	52	4	13 (25%)	29 (56%)	10 (19%)
Backcrossed C57BL/6J F4	48	6	13 (27%)	26 (54%)	9 (19%)

**TABLE 1b:** Observed phenotypic penetrance of *C2cd3*^ex2/ex2^ mutants on CD1 and C57BL/6J backgrounds.

Phenotype	Outcrossed			Backcrossed	
	**CD1**			**C57BL/6J F4**	
	**(*n* = 10)**			**(*n* = 9)**	

Exencephaly	9 (90%)			6 (67%)	
Tight mesencephalic flexure	3 (30%)			3 (33%)	
Abnormal heart looping	3 (30%)			5 (56%)	
Twisted body axis	9 (90%)			7 (78%)	
Pericardial edema	3 (30%)			4 (44%)	

Genetic modifiers have been hypothesized to alter phenotypic presentation associated with mutations in ciliary genes (Reiter and Leroux, 2017). Given the variation in phenotypic penetrance between *Hty* (C3H/HeN background) and *C2cd3^ex2/ex2^* mutants, we examined if background strain contributed to severity and degree of penetrance in *C2cd3^ex2/ex2^* mutants. The *C2cd3^ex2^* line was generated on an outbred CD1 background, and backcrossed over seven generations onto the inbred C57BL/6J background. We observed that embryos with abnormal heart looping, pericardial edema, and tight mesencephalic flexure increased from 30% on a CD1 background to 56, 44, and 33%, respectively on a C57BL/6J:CD1 mixed background, whereas the percentage of embryos showing exencephaly and twisted body phenotypes was reduced (Table 1B, C57BL/6J, F4). The purity of genetic background was examined by miniMUGA (Mouse Universal Genotyping Array) analysis. *C2cd3^ex2/+^* embyros on an outbred CD1 background possessed 28.6% of the potential diagnostic alleles for C57BL/6J (Table 2). In subsequent crosses on the C57BL/6J background, approximately 97% of the potential diagnostic alleles were observed in F2 mice and 98% in F7 mice.

**TABLE 2:** Percentage of diagnostic alleles present in *C2cd3^ex2/+^* mice on CD1 and C57BL/6J backgrounds.

Background	C57BL/6J substrain, %
CD1	28.6
CD1;C57BL/6J, F2	97.4
CD1;C57BL/6J, F7	98.1

In light of these variable phenotypic presentations, we examined C2CD3 protein expression in both control and *C2cd3^ex2/ex2^* mutants on C57BL/6J and CD1 backgrounds. Lysates from E9.5 whole embryos of C57BL/6J or CD1 background revealed that the expression of the 255 kDa isoform was lost in mutant embryos on both genetic backgrounds, while the 205 kDa isoform was maintained (Figure 2D, top and bottom asterisks, respectively). Interestingly, 232 kDa isoform was undetectable likely because the lysate was collected from whole embryos instead of specific tissue. As deletion of primary cilia often results in aberrant Gli protein processing, we also assayed for Gli3 full length (Gli3FL) and Gli3 repressor (Gli3R) expression in *C2cd3^ex2/ex2^* mutants. While *C2cd3^ex2/ex2^* mutants on either background exhibited impaired Gli3 processing, the total amount of Gli3 protein was more robust in embryos on the CD1 background and the ratio between Gli3FL and Gli3R was higher on C57BL/6J background (Figure 2D and Supplementary Figure 2). Thus, variation in Gli3 protein expression and the Gli3FL/R ratio could contribute to changes in expressivity of phenotypes as the mutation was backcrossed onto a C57BL/6J background.

### *C2cd3* Is Required for Craniofacial Development

The majority of craniofacial malformations associated with mutations in C2cd3 and OFD14 stem from neural crest derived tissues. Early embryonic lethality in *C2cd3^ex2/ex2^* mutants prevented an indepth examination of neural crest and craniofacial development. To better understand the function and processing of *C2cd3* relative to craniofacial development, we generated two conditional murine mutants targeting distinct protein domains, and crossed them to neural crest specific drivers.

**FIGURE 3:**
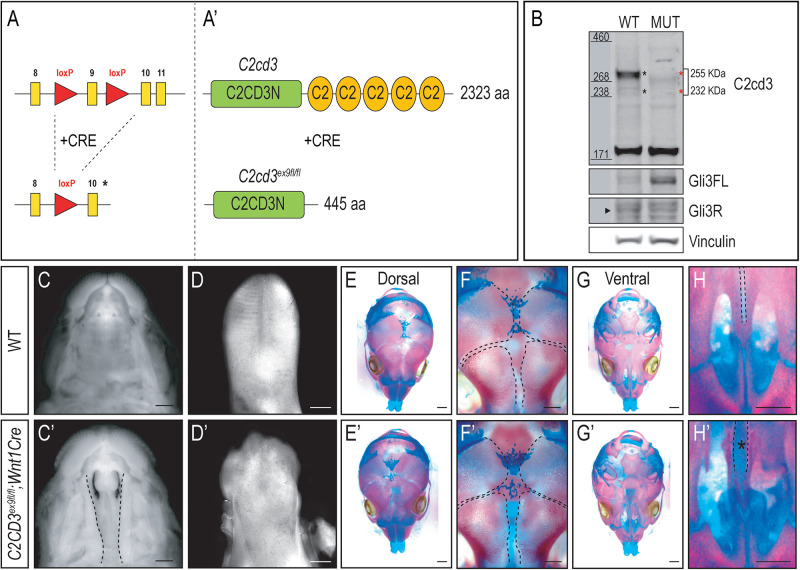
Conditional loss of PKC-C2 domains of C2cd3 results in craniofacial phenotypes. **(A,A′)** Schematic of conditional mouse line generated by CRISPR-targeting with LoxP sequences flanking exon 9. **(B)** Western blot of C2cd3 expression in E11.5 facial prominences showing deletion of the 255 and 232 kDa isoforms in and *C2cd3^ex9fl/fl^;Wnt1-Cre* embryos (red asterisks, *n* = 4 for both wild type and mutant, not shown). **(C,C′)** Palatal and **(D,D′)** glossal views of wild-type and *C2cd3^ex9fl/fl^;Wnt1-Cre* E15.5 embryos. Cleft palate [**(C′)**, dotted lines] and dysmorphic tongue are present in *C2cd3^ex9fl/fl^;Wnt1-Cre* embryos. **(E–H′)** Dorsal and ventral views of whole mount skeletal staining reveals delayed ossification of palatine bone in *C2cd3^ex9fl/fl^;Wnt1-Cre* embryos [**(H,H′)**, asterisk]. Scale bars: 0.5 mm.

As the name implies, C2cd3 contains an array of five classical PKC-C2 domains which are predicted to be involved in targeting proteins to the cell membrane. Despite the presence of these domains, there has been very little exploration into their distinct role relative to C2CD3 function, specifically during craniofacial development. To examine the role of PKC-C2 domains, we generated a conditional mouse line in which LoxP sequences flanked exon 9 (*C2cd3^ex9–flox^*, Figures 3A,A′). Exon 9 was chosen because upon Cre recombination, all PKC-C2 domains would be excised, generating a 445 aa truncated protein. Moreover, all the *C2cd3* splice variants containing exon 9 were recombined when Cre was expressed. Western blot analysis in wild-type and *C2cd3^ex9fl/fl^;Wnt1-Cre* embryos confirmed the loss of the 255 and 232 kDa isoforms in E11.5 mutant facial prominences (Figure 3B). Mutant facial prominences also exhibited increased Gli3FL/Gli3R ratio (Figure 3B), indicating the cilia were functionally impaired. *C2cd3^ex9fl/fl^;Wnt1-Cre* mutant mice survived until late gestation (E18.5) and presented with phenotypes common in OFD14, including cleft palate and a dysmorphic tongue (Figures 3C–D′). Whole mount skeletal staining as well as microCT analysis of E17.5 embryos did not show defects in frontal bone or frontal suture formation (Figures 3E–F′ and Supplementary Figure 4), but revealed delayed ossification of palatine bone in *C2cd3^ex9fl/fl^;Wnt1-Cre* mutant embryos (Figures 3G–H′).

**FIGURE 4:**
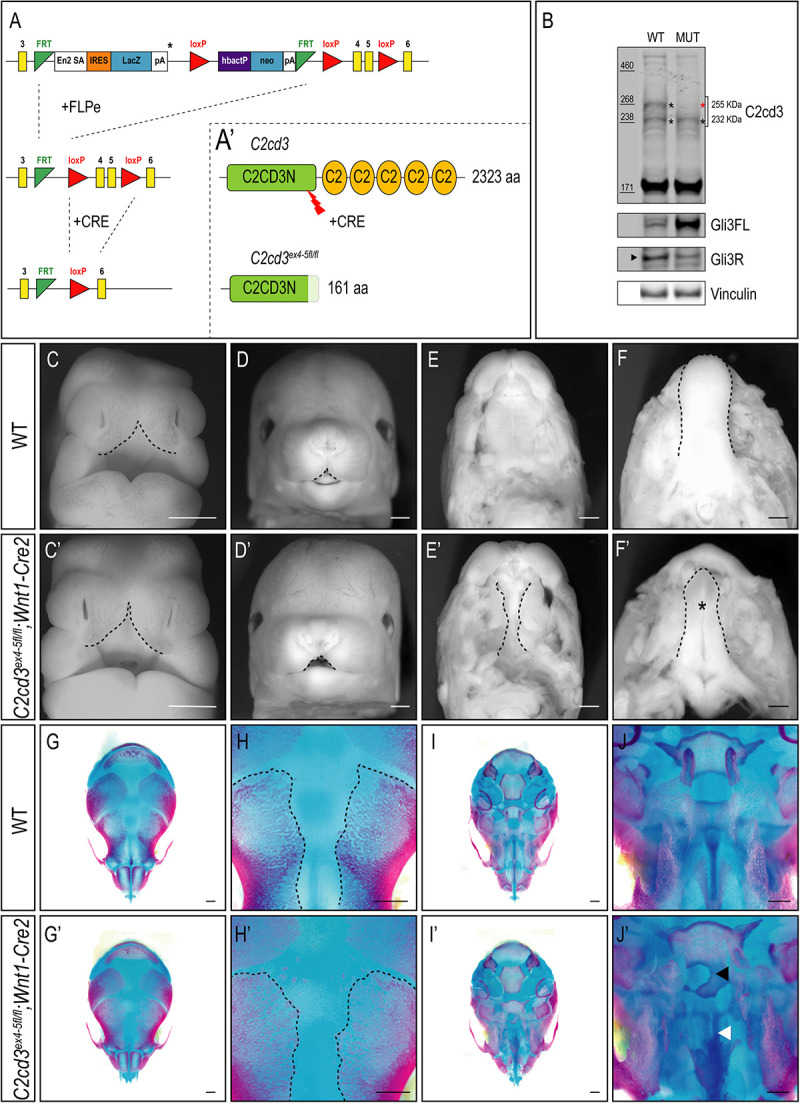
Conditional loss of C2CD3N-C2 domain results in craniofacial phenotypes. **(A,A′)** *C2cd3*^LacZ^ mice were bred to FLPeR mice to excise the LacZ expression cassette, resulting in *C2cd3^ex4– 5flox^* progeny with LoxP sequences flanking exon 4 and 5 and generating a protein with a truncated C2CD3N-C2 domain. **(B)** Western blot of C2cd3 expression in E11.5 facial prominences showing deletion of the longest C2cd3 isoform 255 kDa in and *C2cd3^ex4– 5fl/fl^; Wnt1-Cre* (red asterisk, *n* = 4 for both wild type and mutant, not shown). **(C,C′)** *C2cd3^ex4– 5fl/fl^;Wnt1-Cre2* embryos have characteristic midline widening (dotted line) at E11.5 compared to wild-type, which is exacerbated at E15.5 [**(D,D′)**, dotted lines], as well as cleft palate [**(E,E′)**, dotted line] and a hypoplastic tongue [**(F,F′)**, asterisk]. **(G,G′)** Dorsal views of skull. E18.5 *C2cd3^ex4– 5fl/fl^;Wnt1-Cre2* embryos have normal frontal bone and frontal suture **(H,H′)** development. **(I,I′)** Ventral views of skull. E18.5 *C2cd3^ex4– 5fl/fl^;Wnt1-Cre2* embryos present with abnormal bone growth of the sphenoid [**(J,J′)**, black arrowhead] and palatine bone [**(J,J′)**, white arrowhead]. Scale bars: 0.5 mm.

In addition to the array of PKC-C2 domains, C2cd3 also contains a divergent C2CD3N-C2 domain at the N-terminus of the protein. While this domain is conserved among various species (Zhang and Aravind, 2012), the function of C2CD3N-C2 domain remains completely unknown. To determine the role of the C2CD3N-C2 domain, *C2cd3^LacZ^* mice were bred to FLPeR mice (Farley et al., 2000) to excise the LacZ expression cassette (Figure 4A). The resulting progeny (*C2cd3^ex4–5–flox^*), in which LoxP sequences flanked exon 4 and 5, generated a protein with a truncated C2CD3N-C2 domain (Figure 4A′). To assess the C2CD3N-C2 domain in neural crest cells, we utilized the *Wnt1-Cre2* driver (Lewis et al., 2013) and assayed protein expression in facial prominences of E11.5 embryos via Western Blot analysis. Relative to wild-type embryos, the 255 kDa isoform of C2cd3 was lost in *C2cd3^ex4–5fl/fl^;Wnt1-Cre2* facial prominences. Interestingly, the 232 kDa isoform remained detectable in mutants (Figure 4B), suggesting that the 255 kDa isoform is required craniofacial development. Typical of many ciliopathic mutants, *C2cd3^ex4–5fl/fl^;Wnt1-Cre2* embryos also displayed an increased Gli3FL/Gli3R ratio (Figure 4B). *C2cd3^ex4–5fl/fl^;Wnt1-Cre2* mutants survived until late gestation (E18.5). Contrary to *C2cd3^ex9fl/fl^;Wnt1-Cre* mutants which didn’t reveal a phenotype until approximately E15.5, *C2cd3^ex4–5fl/fl^;Wnt1-Cre2* phenotypes were detected as early as E11.5, with characteristic midline widening (Figures 4C,C′, dotted line), which was exacerbated by E15.5 (Figures 4D,D′, dotted line). *C2cd3^ex4–5fl/fl^;Wnt1-Cre2* mutants also presented with cleft palate (Figures 4E,E′, dotted line) and a hypoplastic tongue (Figures 4F,F′, asterisk). Thus, relative to *C2cd3^ex9fl/fl^;Wnt1-Cre*, *C2cd3^ex4–5fl/fl^;Wnt1-Cre2* mutants had an earlier onset of craniofacial phenotypes that were more severe in nature. Whole mount skeletal staining of E18.5 *C2cd3^ex4–5fl/fl^;Wnt1-Cre2* mutants did not reveal defects in frontal bones or frontal suture (Figures 4G–H′), but exhibited delayed ossifications in sphenoid (Figures 4J,J′, black arrowhead) and palatine bones (Figures 4J,J′, white arrowhead) near the cranial base (Figures 4I–J′). These results suggested that the C2CD3N-C2 domain also plays a functional role during neural crest and craniofacial development, distinct from PKC-C2 domains.

## Discussion

Ciliopathies represent a growing disease class with a significant impact on craniofacial development. As no treatments are currently available for these disorders, gaining increased mechanistic understanding of their etiology through model systems is vitally important. Herein, we report three novel animal model systems to allow for future mechanistic studies regarding the onset of ciliopathic phenotypes. Our initial analyses revealed that *C2cd3* was expressed ubiquitously during early embryonic development, and heterogeneously later in development. Robust knockdown of *C2cd3* expression via CRISPR genome editing resulted in severe developmental defects culminating in early embryonic lethality. Moreover, the phenotypic penetrance and severity varied according to genetic background. We also demonstrated that the N-terminal C2CD3N-C2 domain was functionally more critical compared to C-terminal PKC-C2 domains during craniofacial development, by examining conditional alleles with two neural crest-specific drivers. These studies not only generate several new resources for the craniofacial community, but also suggest several factors that contribute to the onset of craniofacial phenotypes in ciliopathic conditions.

### Variation in Ciliopathic Phenotypes Is Likely Due to Genetic Modifiers

Variable penetrance and lack of genotype-phenotype concordance has long been a challenge for definitive diagnoses of many genetic diseases. Ciliopathies often exhibit multiple and overlapping clinical features owing to the fact that cilia extend from almost all types of cells in the body. For example, Oral-facial-digital syndrome 1-18 (OFD1-18), short-rib polydactyly syndromes (SRPS), Jeune asphyxiating thoracic dystrophy (JATD), Ellis van Creveld syndrome (EVC), and Cranioectodermal dysplasia (CED) all present with varied and overlapping phenotypes making diagnosis difficult. Yet, despite this understanding, the factors that contribute to variable and overlapping phenotypic presentation remain nebulous.

Studies using murine models on inbred background have provided valuable information as to the factors that contribute to phenotypic variability (Nadeau, 2001; Hamilton and Yu, 2012; Kousi and Katsanis, 2015). Numerous studies have reported different levels of phenotypic severity on various inbred backgrounds (Jones et al., 2008), as utilization of these strains can more readily identify genetic modifiers via SNP array, GigaMuga and quantitative trait locus (QTL) analysis (Morgan et al., 2015; Snedeker et al., 2019). Studies such as these have been instrumental in identifying genetic modifiers that can contribute to increasing the variability of phenotypic presentations in ciliopathies. One example of evidence of such modifiers was the report demonstrating that a 430C to T transition in *MGC1203* gene (a pericentriolar protein CCDC28B that interacts with several Bardet–Biedl syndrome, BBS proteins) has an epistatic effect on *BBS* patients (Badano et al., 2006). While mutations in *MGC1203* were insufficient to cause BBS, individual BBS patients who carry the 430T variant of *MGC1203* were more severely affected and have early onset retinitis pigmentosa. Another example of genetic modifiers impacting ciliopathic phenotypes was demonstrated via the relationship between *AHI1* and *NPHP1*. Both *AHI1* and *NPHP1* encode ciliary proteins that physically interact, and mutations found in these two genes are associated with Joubert syndrome. Interestingly, specific variants in these genes significantly increased the risk for onset of tissue-specific phenotypes in patients (Louie et al., 2010).

Although it remains a challenge to identify genetic modifiers for disease-linked genes in humans, murine models may provide useful insight into origins of phenotypic variation. Our data indicated that the severity of phenotypic presentations in *C2cd3^ex2/ex2^* mutant mice is variable on different genetic background (Table 1B), suggesting there are potential genetic modifiers for C2cd3. As C2cd3 localizes to the distal end of the mother centriole and interacts with other centriolar proteins in a hierarchical manner, it is possible that any variant within proteins that localize to that area could impact phenotypic severity of C2cd3 mutations. Furthermore, hypotheses such as these could also apply to variation in human OFD patients, in terms of phenotypic presentation, diagnostic assessment and clinical outcome.

### Functional Roles of *C2cd3* Splice Variants and Isoforms

Most genes have multiple exons, which can contribute to specific isoforms via alternative splicing. Exons are defined by the 5′ splice site, the 3′ splice site and the branch point. These sequence elements can be recognized by the spliceosome complex, which undergoes serial events to remove the introns of pre-mRNA and to form mature mRNA. In general, exons that are used and included alternatively in the mature mRNA, have splice sites and branch point that deviate moreso than the consensus sequences present in constitutively used exons; hence, they have lower binding affinity for the spliceosome. From an evolutionary point of view, alternative splicing is used to increase diversity under different physiological conditions to affect function, binding affinity, and localizations of the protein products (Kelemen et al., 2013). Murine *C2cd3* has 33 exons and 8 splice variants.^2^ Among these variants, seven are protein-coding, producing four distinct protein isoforms (255, 232, 214, and 205 kDa), while one is a non-coding mRNA (Supplementary Figure 1). Our data revealed that while *C2cd3* was expressed ubiquitously in early developmental stages, the level of expression and the prevalence of distinct isoforms varied between different tissues. Given the variable temporal and spatial expression of *C2cd3* during craniofacial development, it will be important to understand the distribution and spatial expression of individual splice variants/protein isoforms over time. Our data herein suggested that the N-terminal C2CD3N-C2 domain was more critical during craniofacial development than C-terminal PKC-C2 domain. Interestingly, within the four C2cd3 protein isoforms there are two isoforms that contain a C2CD3N-C2 domain (255 and 214 kDa; Supplementary Figures 1A,C). We hypothesize that these two isoforms are expressed in tissues most affected in *C2cd3^ex2/ex2^* or *C2cd3^ex4–5fl/fl^;Wnt1Cre2* mutants, in which the targeted mutations disrupt C2CD3N-C2 domain. Alternatively, we hypothesize that isoforms without a C2CD3N-C2 domain (232 and 205 kDa; Supplementary Figures 1B,D) are dispensable for craniofacial development, as the 232 kDa isoform is still expressed *C2cd3^ex4–5fl/fl^;Wnt1Cre2* facial mesenchyme. Our future studies will focus on the spatial expressions and specialized functions for each C2cd3 isoform expressed in craniofacial tissues.

### A Role for the C2CD3N-C2 Domain During Ciliogenesis and Protein–Protein Interaction

Many ciliary proteins in the transition zone contain both classical PKC-C2 and novel C2-domains. These domains have been hypothesized to serve as a functional interaction module within the transition zone (Zhang and Aravind, 2010, 2012; Remans et al., 2014). Although the classical PKC-C2 domain contains a Ca^2+^-binding pocket, which makes contact with the plasma membrane, all other C2 domains within the superfamily do not bind Ca^2+^ (Zhang and Aravind, 2010). Moreover, novel C2 domains, including the C2CD3N-C2 domain, have a unique sequence and are highly conserved among different orthologs, yet share little sequence homology to other known C2 domains. For example, a recent study reported that centriolar protein CEP120 possessed three Ca^2+^-independent, novel C2 domains that potentially played a role in mediating microtubule-binding (Sharma et al., 2018). Our data provide further evidence for the functional importance of a non-classical C2 domain, specifically within craniofacial development. Disruption of C2CD3N-C2 domain in *C2cd3^ex4–5fl/fl^;Wnt1Cre2* mice resulted in severe craniofacial phenotypes including midfacial widening, palatal clefting and hypoglossia; while deletion of all PKC-C2 domains in *C2cd3^ex9fl/fl^;Wnt1Cre* mice produced only mild craniofacial phenotypes. Interestingly, it has been shown that C2CD3 ortholog SAS-1 in *C. elegans* contains a unique N-terminal, non-classical C2 domain (von Tobel et al., 2014). SAS-1 also localizes to the centriole and has the ability to bind to and stabilize microtubules. Based on these results, we hypothesize that the C2CD3N-C2 domain may have a distinct and required function when compared to the five PKC-C2 domains, specifically in microtubule binding/stabilization, in addition to the known functions in membrane targeting and vesicle docking. Our future studies will address the specific role of the C2CD3N-C2 domain within tissues of the craniofacial complex.

### Functional Importance of Ciliary Proteins in Skeletogenesis

In addition to axial skeletal defects, patients with ciliopathies also frequently present with malformation of the craniofacial skeleton (Baker and Beales, 2009; Schock and Brugmann, 2017). The importance of cilia in skeletal development has been demonstrated in many animal models (Yuan and Yang, 2015). Here we show that deletion of *C2cd3* within the progenitors of the craniofacial skeleton (neural crest cells) affected the development of intramembranous skeletal elements. With deletion of all PKC-C2 domains in C2cd3, we observed delayed ossifications in the cranial base (palatine bones). Moreover, deletion of N-terminal C2CD3N-C2 domains in addition to all PKC-C2 domains extended the delayed ossifications further to the sphenoid bone. This result was consistent with the previous findings that deletion of ciliary genes *Kif3a* or *Ift88* using *Wnt1-Cre* resulted in reduced neural crest cell-derived cranial bones (Kolpakova-Hart et al., 2007; Tian et al., 2017). As the majority of the craniofacial skeleton is derived from cranial neural crest cells and differentiates through intramembranous ossification, it will be important to understand how deletion of C2cd3 impacts the sequential steps of intramembranous ossification including neural crest condensation, osteoblast/osteocyte differentiation, and matrix/periosteum formation. Our future work will specifically address this question and examine the molecular basis for the onset of these phenotypes.

Mouse models established here can serve as powerful tools to study the function of C2cd3 and primary cilia during development of the craniofacial skeleton. Ciliopathy patients with craniofacial anomalies often have abnormal skeletogenesis and require surgical reconstructions using autologous grafts from mesodermal-derived bones like the rib or iliac crest. The surgical process is painful and often the tissue fails to engraft. Using tools like those described herein, we are able to begin understanding cellular and molecular mechanisms necessary for skeletogenesis within the craniofacial complex and potentially apply this knowledge toward the application of alternative surgical repair for OFD, as well as ciliopathy patients, in general.

## Materials and Methods

### Mice

Mouse ES cell line *C2cd3^TM 1a(EUCOMM)Wtsi^* was purchased from EuMMCR.^3^ ES cell microinjection was performed by Transgenic Animal and Genome Editing (TAGE) Core facility of Cincinnati Children’s Hospital Medical Center (CCHMC). The resulting chimeric mice were bred to wild type to obtain heterozygous (*C2cd3*^LacZ/+^) animals. CRISPR-targeted *C2cd3* knockout mice were designed and generated by TAGE Core facility of CCHMC. All animals were maintained by Veterinary Services of CCHMC with IACUC approval.

### Analysis of Mutant Embryos

C2cd3 mice were maintained on a CD1 background or serially backcrossed to C57BL/6J. Genotyping for the 8 bp deletion was performed by PCR and Sanger Sequencing. Embryos were harvested via Caesarian section, dissected, and examined. Embryos were fixed in Bouin’s fixative overnight at room temperature, washed 3 times with PBS, and imaged on the Leica M165FC microscope.

### X-Gal Staining

*C2cd3*^LacZ/+^ embryos at various time points were dissected in cold PBS (pH 7.4) and fixed with 2% Paraformaldehyde (PFA) plus 0.25% Glutaraldehyde, 0.02% NP-40, 0.1% Sodium Deoxycholate in PBS at 4°C with gentle shaking for 1-2 hours. The samples were then switched to X-gal staining solution (5 mM K_3_Fe(CN)_6_, 5 mM K_4_Fe(CN)_6_^∗^3H_2_O, 2 mM MgCl_2_, and 1 mg/mL X-gal in PBS) at 37°C with gentle shaking for 12-16 hours. The staining was terminated with 3% DMSO in PBS and post-fixed by 4% PFA in PBS. Whole mount images were taken before obtaining microtome sections.

### Western Blot

Protein lysate was prepared from E9.5 mouse embryos or E11.5 facial prominences (epithelium was removed by treating the samples with 3 mg/mL Dispase/PBS at 37°C for 30 min) in RIPA buffer with protease and phosphatase inhibitors and 20 μg of sample was loaded onto 3–8% NuPAGE Tris-Acetate gels (ThermoFisher) for Western blot. C2cd3 (1:500; Antibodies-online.com ABIN2591132), Gli3 (1:1,000; R&D Systems, AF3690), and Vinculin (1:2,000; Santa Cruz, sc-73614) antibodies were used. Blots were imaged on LICOR Odyssey imager.

### Immunofluorescence Staining

Cryosections of E9.5 embryos were fixed in 4% PFA for 5 min, washed three times with PBS. The sections were then blocked in 5% normal goat serum in PBS for 30 min and then incubated with Arl13b (1:1,000; Proteintech, 17711-1-AP) and gamma-Tubulin (1:1,000; Sigma, T6557) in blocking buffer at 4°C overnight. Goat anti-rabbit Alexa 488 and goat anti-mouse Alexa 594 were used for secondary antibodies at 1:1,000 in blocking buffer, room temperature, 1–2 h. DAPI (1:10,000) was used for nuclear staining.

### MiniMUGA Genotyping

MiniMUGA (Mouse Universal Genotyping Array genotyping, Neogen Genomics Co.) genotyping was performed by GeneSeek (Neogen; Lincoln, NE, United States). Genomic DNA was isolated using ThermoFisher PureLink gDNA kit. Approximately 10,800 markers were analyzed with MiniMUGA.

## Data Availability Statement

The datasets presented in this study can be found in online repositories. The names of the repository/repositories and accession number(s) can be found in the article/Supplementary Material.

## Ethics Statement

The animal study was reviewed and approved by Veterinary Services of CCHMC with IACUC approval.

## Author Contributions

C-FC performed all embryonic and expression analysis. KB and YY performed all backcrossing studies and genetic background analysis. SB conceived and funded project. SB, C-FC, and KB wrote and edited manuscript. All authors contributed to the article and approved the submitted version.

## Conflict of Interest

The authors declare that the research was conducted in the absence of any commercial or financial relationships that could be construed as a potential conflict of interest.

## Footnotes

1 https://www.omim.org

2 https://www.ncbi.nlm.nih.gov/gene/277939

3 https://www.eummcr.org

## Supplementary Material

Supplementary Figure 1 Reported splice variants in murine C2cd3. Seven protein-coding variants (NCBI accession number NM or XM) produce four protein isoforms (one non-coding mRNA is also predicted (XR_003946487.1, not shown)). Two isoforms contain C2CD3N-C2 domain **(A,C)** while two isoforms contain only PKC-C2 domains **(B,D)**.

Supplementary Figure 2 **(A)** Western blot of Gli3FL and Gli3R in CD1 and C57BL/6J backgrounds. **(B)** Densitometry of Gli3FL and Gli3R in each lane was measured by ImageJ. Gli3FL/Gli3R ratio is higher on C57BL/6J background (*n* = 4, *t*-test, ^∗^ indicates *P* < 0.05).

Supplementary Figure 3 Exon expression in *C2cd3* mutants. **(A)** RT-qPCR of exon 8-9 and 32-33 in wild-type (*n* = 5), *C2cd3*^ex2/+^ (*n* = 6), and *C2cd3*^ex2/ex2^ (*n* = 4) embryos shows significantly reduced, but detectable levels of *C2cd3* downstream of exon 2 (*t*-test, ^∗^ indicates *P* < 0.05; ^∗∗^ indicates *P* < 0.01; NS: not significant).

Supplementary Figure 4 MicroCT analysis of E17.5 mouse embryos. Snapshots of microCT 3D images of **(A,A′)** wild type and **(B,B′)** *C2cd3^ex9fl/fl^;Wnt1Cr*e mutant embryos. The frontal bones **(A,B)** and mandibular bones **(A′,B′)** are marked by blue. The bone volume of **(C)** frontal bones and mandibular bones **(D)** was measured by Inveon Workstation Software (Siemens Medical Inc), the results indicates that there is no significant difference between wild type and mutant (*n* = 4 for wild type or mutant, *t*-test, NS: not significant).
