# Supplementary figures and images for: Centriolar Protein C2cd3 Is Required for Craniofacial Development
Source: Front Cell Dev Biol. 2021 Jun 15;9:647391. doi: 10.3389/fcell.2021.647391 (PMC8239364; doi:10.3389/fcell.2021.647391)

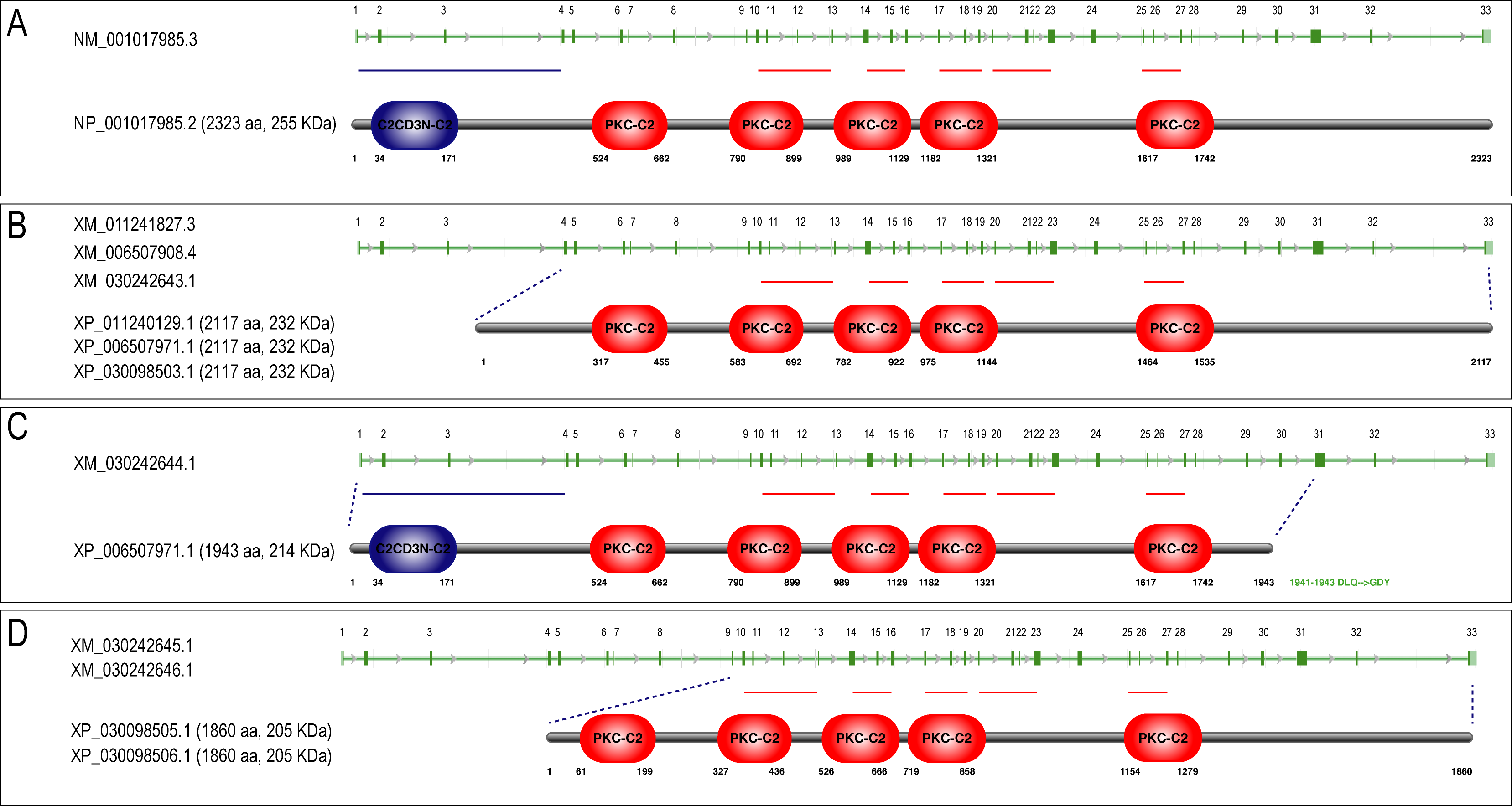

Supplement: Supplementary Figure 1 — Reported splice variants in murine C2cd3. Seven protein-coding variants (NCBI accession number NM or XM) produce four protein isoforms (one non-coding mRNA is also predicted (XR_003946487.1, not shown)). Two isoforms contain C2CD3N-C2 domain (A,C) while two isoforms contain only PKC-C2 domains (B,D). [file Image_1.TIF]

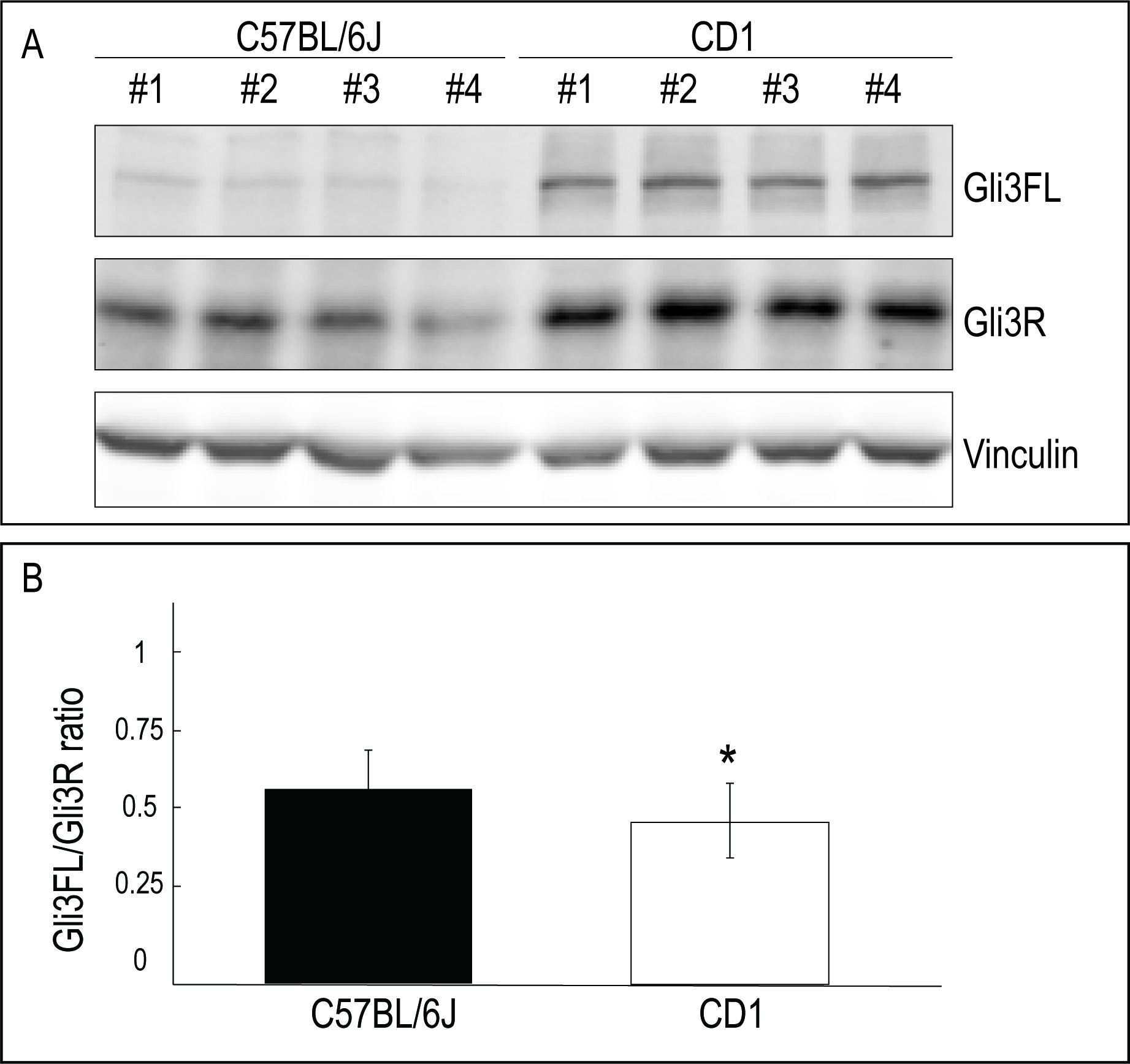

Supplement: Supplementary Figure 2 — (A) Western blot of Gli3FL and Gli3R in CD1 and C57BL/6J backgrounds. (B) Densitometry of Gli3FL and Gli3R in each lane was measured by ImageJ. Gli3FL/Gli3R ratio is higher on C57BL/6J background (n = 4, t-test, ∗ indicates P < 0.05). [file Image_2.TIF]

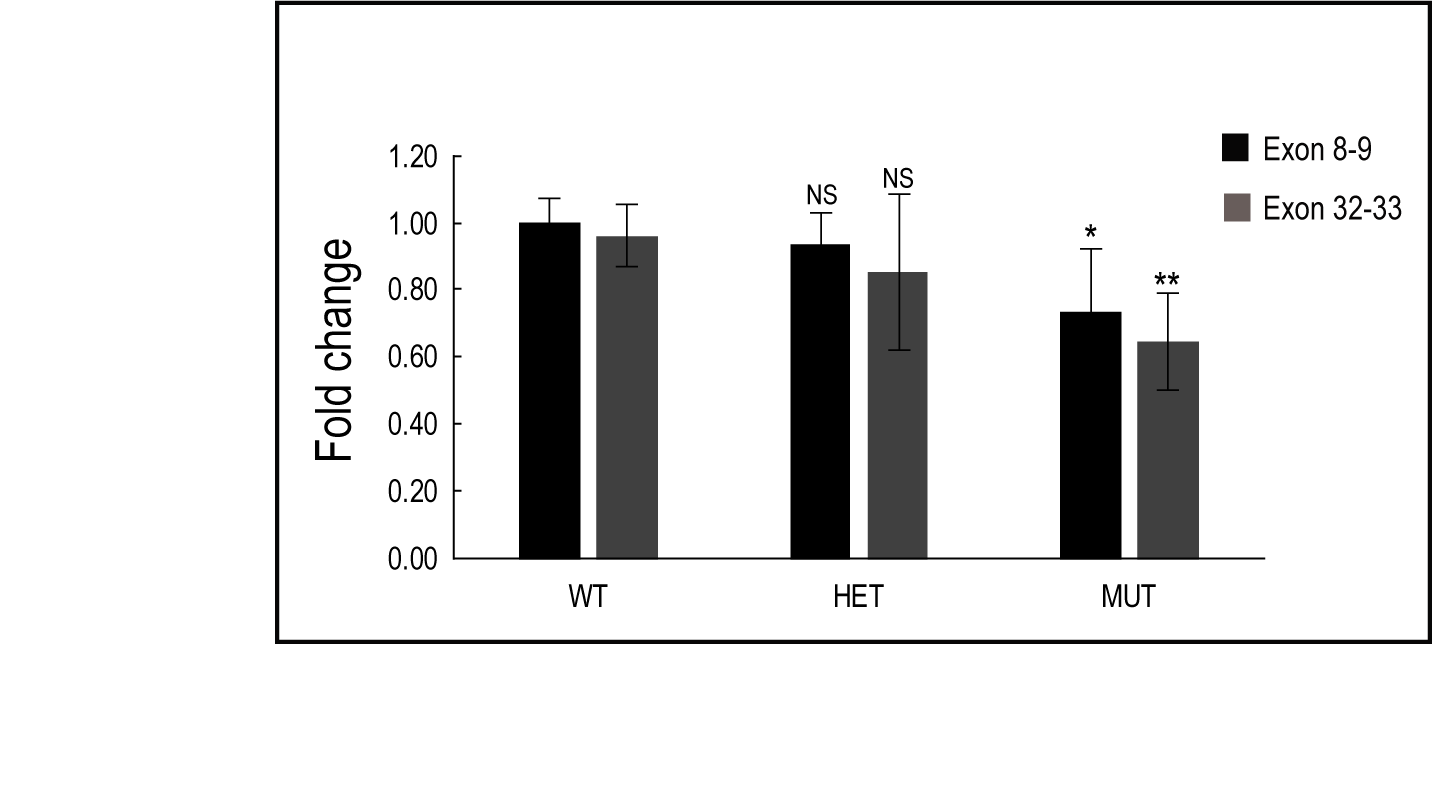

Supplement: Supplementary Figure 3 — Exon expression in C2cd3 mutants. (A) RT-qPCR of exon 8-9 and 32-33 in wild-type (n = 5), C2cd3ex2/+ (n = 6), and C2cd3ex2/ex2 (n = 4) embryos shows significantly reduced, but detectable levels of C2cd3 downstream of exon 2 (t-test, ∗ indicates P < 0.05; ∗∗ indicates P < 0.01; NS: not significant). [file Image_3.TIF]

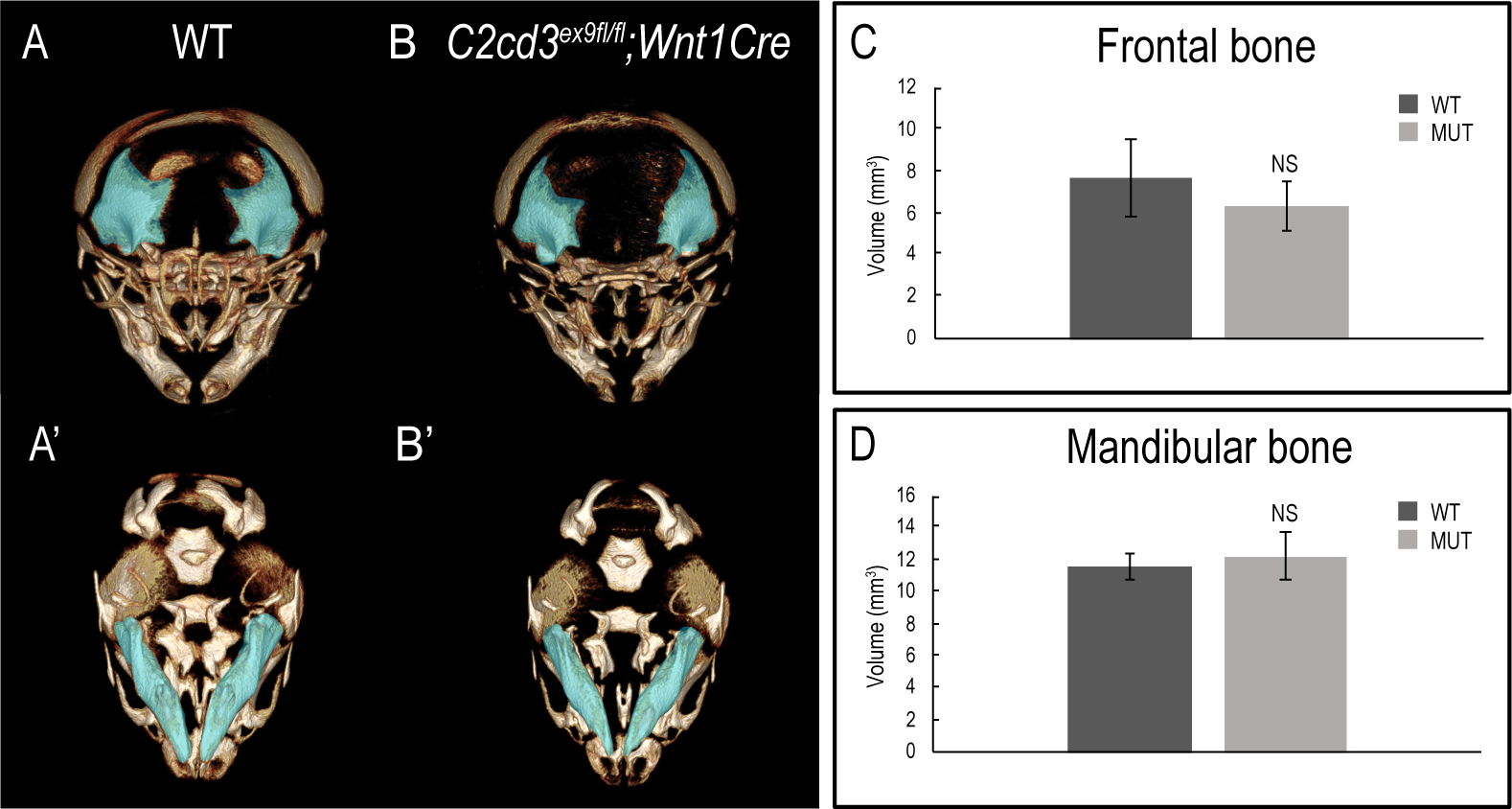

Supplement: Supplementary Figure 4 — MicroCT analysis of E17.5 mouse embryos. Snapshots of microCT 3D images of (A,A′) wild type and (B,B′) C2cd3ex9fl/fl;Wnt1Cre mutant embryos. The frontal bones (A,B) and mandibular bones (A′,B′) are marked by blue. The bone volume of (C) frontal bones and mandibular bones (D) was measured by Inveon Workstation Software (Siemens Medical Inc), the results indicates that there is no significant difference between wild type and mutant (n = 4 for wild type or mutant, t-test, NS: not significant). [file Image_4.TIF]
